# Perceptions about exposure to pesticides among rural school students: identified controversies

**DOI:** 10.1590/0034-7167-2022-0101

**Published:** 2023-02-06

**Authors:** Simone Domingues Garcia, Dulce Maria Strieder

**Affiliations:** I Universidade Estadual do Oeste do Paraná. Cascavel, Paraná, Brazil

**Keywords:** Science Teaching, Health, Pesticides, Public Policy, Nursing, Enseñanza de Ias Ciencias, Salud, Pesticidas, Políticas Públicas, Enfermería, Ensino de Ciências, Saúde, Agrotóxicos, Políticas Públicas, Enfermagem

## Abstract

**Objectives:**

to identify, according to students’ perception, controversies related to the term “pesticide” and its influence on health.

**Methods:**

field research, with qualitative analysis via discursive textual analysis. Collective interviews were carried out with students of ninth grade of elementary school from four rural schools in the western region of Paraná.

**Results:**

when analyzing students’ statements, it was identified that pesticides are part of their reality and that of the local community Controversies related to pesticides and health arose, with emphasis on the form of production and the understanding of the real harm generated by exposure and use of pesticides.

**Conclusions:**

approaching pesticides in the context of education and health is essential so that there is a strengthening of discussions, in a critical and contextualized way, in school spaces, expanding the look at the topic as a way of enriching understandings and discourses on the subject.

## RESUMO

**Objetivos:**

identificar, na percepção de estudantes, as controvérsias relacionadas ao termo “agrotóxico” e sua influência na saúde.

**Métodos:**

pesquisa de campo, com análise qualitativa via análise textual discursiva. Foram realizadas entrevistas coletivas com alunos do nono ano do ensino fundamental de quatro escolas do campo da região Oeste do Paraná.

**Resultados:**

ao analisar as falas dos estudantes, identificou-se que os agrotóxicos fazem parte da realidade deles e da comunidade local. Surgiram controvérsias relacionadas aos agrotóxicos e saúde, com destaque para a forma de produção e a compreensão dos reais malefícios gerados pela exposição e uso dos agrotóxicos.

**Conclusões:**

abordar os agrotóxicos no contexto da educação e saúde é essencial para que haja um fortalecimento das discussões, de forma crítica e contextualizada, nos espaços escolares, ampliando o olhar para a temática como forma de enriquecer as compreensões e os discursos sobre o tema.

## RESUMEN

**Objetivos:**

identificar, en la percepción de los estudiantes, Ias controversias relacionadas con el término “pesticida” y su influencia en la salud.

**Métodos:**

investigación de campo, con análisis cualitativo a través del análisis textual discursivo. Se realizaron entrevistas colectivas con alumnos del noveno año de Ia enseñanza fundamental de cuatro escuelas rurales de la región occidental de Paraná.

**Resultados:**

al analizar las declaraciones de los estudiantes, se identificó que los plaguicidas son parte de su realidad y de la comunidad local. Surgieron controversias relacionadas con los plaguicidas y la salud, con énfasis en la forma de producción y la comprensión del daño real generado por Ia exposición y uso de plaguicidas.

**Conclusiones:**

abordar los plaguicidas en el contexto de Ia educación y la salud es fundamental para que haya un fortalecimiento de las discusiones, de forma crítica y contextualizada, en los espacios escolares, ampliando Ia mirada sobre ei tema como forma de enriquecer comprensiones y discursos sobre el tema.

## INTRODUCTION

Brazil remains one of the largest consumers of pesticides in the world, with a significant representation of agriculture in the country’s economic power. However, there is a lack of data on the subject, such as the different types of pesticides used in Brazilian municipalities, together with the lack of knowledge of their real toxic potential, which favors the concealment and invisibility of this important public health concern^(1)^.

The issue of pesticides has been controversial, because, on the one hand, there are those who defend their use in a “controlled” way, and indicate the impossibility of planting, on a large scale, without the use of pesticides, but, on the other hand, there are those who point to the possibility of producing food for the population without the use of pesticides based on a new model of agrarian development^(2)^.

The negative impacts of pesticide use on the environment and human health are increasingly debated and contextualized in the scientific field. It is stated that exposure to pesticides in their chronic form can be associated with diseases in the metabolic, reproductive, endocrine systems and in the increase in cases of different types of cancers. Farmers and family members represent the groups with the greatest damage to health, due to their continuous and prolonged exposure to these substances; however, it should be considered that the entire population can develop chronic effects from cumulative exposure through ingested water and consumption of contaminated food^(3)^.

In citing pesticides and health education, there is a predominance of biomedical discourse, with emphasis on the disease process, even with the existence of public policies aimed at health promotion and prevention. The need for a task force between fields of knowledge, teaching and research institutions is highlighted so that the approach to health, in its broad concept, as access to cultural, leisure and communication goods, and also the well-being physical, mental, social and collective being, is valued in fundamental places, such as schools^(2)^.

With this, pesticides, due to the diversity of approaches, they are a controversial topic, supported by the concept that socio-scientific controversies are characterized by the generation of doubts, both in the scientific community and in society, such as: environmental, social and cultural impacts caused by new technologies, such as transgenic crops, nanotechnologies, biotechnology incursions on the borders of nanotechnologies and pesticides^(4)^.

Finally, schools represent a fundamental space in the construction of knowledge and in the practice of health education, and the approach of controversial and significant topics for society, such as pesticides^(3)^, is relevant.

The present study is justified by viewing the nurse as a key tool for the development of health education in school spaces, giving new meaning to the school as a scenario for health promotion and important debates on topics experienced by the community, with the appreciation of its potential for producing citizenship and changes in the way of life.

## OBJECTIVES

To identify, according to students’ perception, controversies related to the term “pesticide” and its influence on health, together with the relationships established with the social, economic, environmental and cultural contexts experienced.

## METHODS

### Ethical aspects

The research was approved by the Research Ethics Committee of the *Universidade Estadual do Oeste do Paraná* (UNIOESTE). Underage students needed the authorization of their parents or guardians to take part in the research, being necessary their signature in the Informed Consent Form (ICF) before the research.

### Study design

This is a descriptive field research with qualitative analysis. In field research, the object/source is approached in its own environment. Data construction is done under the natural conditions in which the phenomena occur, being directly observed without intervention and handling by the researcher. Descriptive and explanatory studies seek to describe the studied characteristics of objects, surpassing the recording and analysis of the studied phenomena^(5)^. We use the COnsolidated criteria for REporting Qualitative research (COREQ), which represents a comprehensive checklist with important aspects of study method and context, results, analysis and interpretations that covers all necessary components of the project.

### Theoretical-methodological framework

As a methodological theoretical framework, controversies were used to bring elucidations and enable a critical look at the topic of pesticides and health^(6)^. The term “socio-scientific controversy” follows the following criteria: arising from the social impacts of scientific-technological innovations that divide both the scientific community and society in general; allow discussion between two or more parties involved about a given controversy, in which their beliefs, arguments and culture are at stake; and arouse indecision, in which people may find themselves divided, because this reflection involves value judgments that make it impossible to resolve them only through the analysis of evidence or experience^(2)^.

Pesticides can be included in all the definitions presented, since the beginning of their use in Brazil represented a significant social impact, associated with the green revolution and the gains that would arise for the population with the mechanization of the countryside, the large production of food and structural change in rural areas^(1)^. Regarding the discussions, pesticides are linked to the strength of agribusiness, which generates conflicts of ideas in different groups, with the emergence of indecisions and the need for approximations with the topic.

### Study setting

The research was carried out in four state schools in the countryside, selected in western Paraná. The work in this region becomes relevant, considering that the commercialization of pesticides presented the following data in the municipalities of Paraná between 2014 and 2017: Cascavel (2,886.4 tons/year), Tibagi (1,798.1 tons/year), Castro (1,608.6 tons/year), Assis Chateaubriand (1,565.3 tons/year), Toledo (1,458.3 tons/year), Guarapuava (1,414 7 tons/year), Candói (1,409 tons/year), Palmeira (1,252.9 tons/year), Corbélia (1,205.4 tons/year) and Palotina (1,176.7 tons/year). This shows that the region where the study was carried out sells a significant amount of pesticides, and a greater analysis of the factors involved with their use is essential^(7)^.

### Methodological procedures

The interviews were semi-structured with directive questions, with the obtaining of possible categorization answers. The interview gives interviewees greater flexibility and clarity for possible doubts, with greater approximation of the interviewer. They can be performed individually or collectively, with emphasis on collective interviews in the sharing and valorization of dialogues by participants^(8)^.

Students from four state rural schools in the South, Southeast, North and Northeast regions of the municipality of Cascavel, enrolled in the ninth year of elementary school, were included. These groups of students were selected, considering that, in this age group, they can already associate the topic with other factors, such as health, due to the use of pesticides being inserted in their family routine and cultural factors in the region. The identification of interviewees is in parentheses after each speech, with its division according to the regions of the visited schools.

### Data collection and organization

The interviews were recorded and, after use, the data were discarded, with confidentiality of the information contained and following the ethical principles of the research. Groups were formed by students from 13 to 16 years old, with a variation in the number of participants in each group from 11 to 20 students, according to guardians’ acceptance and authorization to participate. The interviews were, on average, 30 to 40 minutes long, and occurred during the period of students’ classes, according to previous contact with the school. They were held in June 2019.

### Data analysis

Data were analyzed from discursive textual analysis (DTA). For DTA to be implemented, the researcher must carry out successive readings and intense movement of interpretation and production of arguments, to contemplate the details identified in the texts, with an eye towards different possibilities and understandings^(9)^.

It is stated that, when using DTA, the research subjects are valued in their modes of expression of the phenomena, by focusing their searches on collective networks of subjectively constructed meanings, which the researcher challenges himself to understand, describe and interpret. DTA is not limited to identified factors, but in the construction and deconstruction carried out during the course of the research, being possible to contemplate social, economic, environmental and cultural contexts throughout the analyzed texts^(9)^.

As this research is part of a doctoral thesis, this article represents one of the aspects investigated, highlighting the controversies related to the topic of pesticides and their relationship with health.

## RESULTS

In order to understand students’ perceptions and identify controversies during the interviews, it is necessary to look at the questions asked. The questions addressed how it was to study and live in that region, if the family worked with agriculture, if they used to help the family and if they knew how to talk about what was produced in that region.

It was asked if students had heard about herbicides, also called pesticides, and, when they thought of pesticides, what were the first words they remembered in addition to the place they heard about the topic.

The question directly related to health was whether they considered that there is any relationship between health and pesticide, with the final question on what they would like to research on pesticide.

## DISCUSSION

When addressing the issue of pesticides, controversies are commonly encountered, and it is possible to identify in the speeches disagreements between the form of production for consumption within the family and the difference in products called organic, as presented by each region identified by the interviewee:

*Yeah, my father plants just for us, but he doesn’t use pesticides.* (Northwest Region)

*Sometimes it doesn’t need to be poisoned.* (Southeast Region)

*That’s how you take care of what you produce at home, so how will you know the care they take like that in the market?* (Southeast Region)

*Not in the garden either.* (East Region)

*In the garden, we don’t use it, at home, no.* (East Region)

*No, in the garden, no, what she consumes, no, my mother didn’t pass on what she was going to sell. She had a vegetable garden, she didn’t pass, so we consumed from that one and she also took it to the fair to sell.* (East Region)

*When it is in the vegetable garden, it is not used as much as it is used in farming* [produced in the region]. (South Region)

*There is a vegetable garden at the school too, there is a vegetable garden, we make the garden and it is all organic, there is no pesticide* (Northwest Region)

*The price is more expensive with organic than with pesticides.* (East Region)

*Normally, organic is always more expensive.* (South Region)

*I know when it’s organic and when it’s not organic because my parents say.* (East Region)

With these statements, it is possible to verify that, when they discuss production for family consumption and at school, there is a concern about not using pesticides, because the products are consumed by family members and acquaintances, unlike when they think about large-scale production. Thus, in relation to controversies, discussions between two or more parties are identified in the face of their arguments and culture^(2)^, since, when referring to the other, there is a distance, without generating concern about the pesticides that are used in products produced in large crops known for monoculture, such as grains, which in students’ eyes are not consumed directly, but used for commerce (such as soybeans and corn) and would be consumed by strangers, but the culture of producing food for their own consumption, such as vegetables, legumes and some types of grains as well, but in smaller quantities, is maintained.

The weakening of the perception of the global (e.g., when considering what is produced on a small and large scale) leads to a weakening of responsibility (each tends to be responsible only for its specialized task) as well as a weakening of solidarity (each of which no longer feels the bonds with its fellow citizens). This becomes a worrying factor, since the isolated look weakens the understanding of society as a whole^(6)^.

In the field of science teaching, pesticides, as a social, cultural and technological product, are seen as a controversial scientific topic, due to the different views sustained in relation to their advantages and disadvantages. On the other hand, especially in the context of rural education, the ethical, environmental and social aspects involved in its use cannot be ignored, especially with regard to commitment of biodiversity and continuity of life of populations: both in the countryside and in the city^(2)^.

When citing the state of Paraná, which includes the schools in the study, it stands out as the third largest consumer of pesticides in Brazil. The total volume of pesticides consumed was 92,160,500 kg in 2016 and 92,398,000 kg in 2017. The state was accounted for 16.23% of Brazil’s grain production in the 2017/2018 harvest, which corresponds to 36,691,400 tons, and the area of 9,734,900 hectares is used for the production of temporary crops, vegetables and crops permanent, according to 2017 data from the Brazilian National Supply Company (CONAB - *Companhia Nacional de Abastecimento*)^(7)^.

Among the difficulties encountered is the accuracy in obtaining total/actual data on the volume and types of pesticides used, contrary to Law 12,527/2011 on access to information. These data are considered relevant for public institutions, researchers, health professionals and society, in order to question and expand the discussion on the topic^(1)^.

When referring to whether a substance used is considered carcinogenic, there is no limit to guarantee that this use will not cause cancer, since the substance itself generates this risk. With the release of its use by the industry, the necessary knowledge on the subject is lacking not only for farmers, but to the entire population and the health team, confusion of symptoms and inadequate treatments are common, as it is not possible to update properly in the face of various changes and new products available. It is essential that health professionals are trained and discussed more on the subject, in order to carry out a diagnosis that critically includes the reality of the person involved, involving a context of health promotion and preservation of the environment^(10)^.

The harmful effects of pesticides, for those who live in rural areas who use them, are commonly related to the final product consumption, such as soy, corn and other grains used for commercialization, underestimating the consequences they can generate on the environment, such as contamination of springs, alteration in the soil, air and in the health of those who live with pesticides and live close to production areas. In a study^(11)^, it was identified that all the farmers interviewed used highly dangerous pesticides and high risk for their own health, and, consequently, for the family, since 81% of the total considered have lived in the rural community for more than 15 years, exposed to pesticides; of these, 59% reported having health problems resulting from the exposure and use of pesticides.

It is noteworthy that the schools that participated in the study did not have a significant distance from the urban region, with tenuous cultural boundaries and similar access to health services, commerce, obtaining water supply, sewage network, internet access, among others, due to geographic proximity and easy displacement.

Thus, controversies are mentioned in which people are divided, because reflection involves value judgments that make it impossible to resolve them only through evidence, analysis or experience^(2)^. In the statements, it is complex for students to understand the risks contained in home and school gardens, even if not using pesticide directly, but due to proximity to areas of large crops.

The term “organic” appeared in some speeches with different references, being associated with the form of production without pesticides, difference in the appearance of food and the value of products offered. This shows that students’ concepts in the classroom differ in view of factors associated with the topic. Organic product consumption is growing more and more in Brazil, associated with sustainable agriculture, natural cultivation and ecological balance, which result in healthier products that respect human beings and the environment. However, consumption is still considered low in the country, due to the lack of information on organic agriculture and its benefits, the high price due to the presence of conventional products, which have more affordable prices on the market, and the lack of availability of products that are produced on a smaller scale^(12)^.

Organic agriculture uses soil conservation techniques, such as crop rotation and crop cultivation, minimum cultivation and green fertilization, with less nutritional loss, contrary to what is observed in conventional agriculture, in addition to avoiding contamination of soils and water resources, which allows the land to remain more fertile and resistant to the attack of parasites. Most organic food production comes from family farming (small farmers), which usually joins an association of cooperative members for the marketing of their products. In addition to a commercially differentiated product, there is a concern with health and the environment, where many of these producers are converting the conventional to organic system, as they understand the need to take care of the ecosystem^(12)^.

Regarding the certification and distribution of organic products in Brazil by region, an increase was identified in the total number of organic producers registered with the Ministry of Agriculture, Livestock and Supply (MAPA) between 2015 and 2017, with the most accentuated expansion in southern states^(13)^. However, it is observed that Brazil still needs policies to encourage organic product consumption. Public policies include the Food Acquisition Program and the Brazilian National School Feeding Program, which also encourage production by family farming^(14)^.

It was reported, as demonstrated by the interviewees, that it is common to use certain pesticides, better known as insecticides in urban areas, in homes, to ward off insects and arachnids, with an association with risk exposure different from the risk offered by the product used in farming.

*My father went to buy a product to apply inside the house, because, as we live here on the farm, we apply as not to see spiders, these animals too, right, is it a type of poison?* (East Region)

*It’s like when our parents buy a product to apply inside the house so it doesn’t get spiders, insects inside the house too.* (East Region)

*At home, it’s just poison, because of the plantations, it’s all poison, because it’s a lot.* (East Region)

*At home, we plant oranges, you know, and we use pesticides to get through because of the insects, that sort of thing.* (East Region)

*It is very harmful to health depending on the chemical.* (South Region)

The statements can be associated with different ways of visualizing pesticides, with differentiation between products for farming and those for domestic use, as if there was a differentiation in the risk of exposure of products and in the understanding of each one’s effect. When referring to risk factor, discussion between two or more parties involved about a given controversy is allowed, in which their beliefs and arguments are at stake^(2)^ as, in this case, the difference between understanding risks and places of exposure.

It is stated that the application of pesticides is probably the only activity in which work production environment contamination is intentional. Pollution is caused in order to combat “crop pests”, whether an herb, a fungus or an insect, considered as “weeds, pests or pests”, which become the target of the action of pesticides, such as herbicides, fungicides or insecticides^(1)^.

Regarding workplace, it was found that, followed by residence, places of exposure for occurrence of intoxication are considered, and residences are also places of risk for the use of these products. When thinking that clothes used during the handling of this product are taken inside the houses with possible splashes, to be later handled for washing, there is a risk of exposure for the other family members^(15)^.

In a study conducted in western Rio Grande do Sul, residences presented other risks, such as irregular storage, non-disposal of empty packages or their reuse and non-provision of Personal Protective Equipment. Storage clearly becomes a critical point in the region, and in the first harvest, 60% of irregularities were identified, and, in the second, this value increased, reaching a total of 67.5%. In the irregular situations found, there are farmers who do not have any isolated place to store pesticides, keeping them in a place with easy access to people and domestic animals^(16)^.

Another factor considered by the respondents in the statements is the way students identify the presence of pesticides, such as:

*You can see when fish dies too.* (Southeast Region)

*The soil becomes drier, more arid.* (Southeast Region)

*One is less harmful to health, one has less pesticides, right, one does not use pesticides. The one here is organic and the other one is not.* (South Region)

Different factors were mentioned in the speeches as a way of witnessing the use of pesticides, such as the change in rivers associated with the death of fish, in the soil and in the food offered, which can be more or less harmful to health. These associations demonstrated the perception that pesticide alters the environment in different ways and that, when questioned, students can cite situations that present important changes and observe the environment in which they live. In this part, controversy can be cited in the reflection in which people involve value judgments, which make it impossible to resolve them only through the analysis of evidence or experience^(2)^.

Visualizing the problem can be uncomfortable, however, for the articulation and organization of knowledge to occur and, thus, to recognize and know the problems of the world, a reform of thought is necessary. However, this reform is paradigmatic, and not programmatic: it is the fundamental issue of education, since it refers to our ability to organize knowledge^(6)^.

Health permeates the speeches in different ways, since, in order to contextualize health, it is necessary to understand it as part of a complexity inserted in a system, with direct and indirect influences from different subjects^(6)^.

In the context of human health, the influence of social, ecological and cultural relationships is recognized to reach a broader concept of health, and, therefore, it is necessary to bring academic groups closer to socio-ecological issues. There is a collective effort to learn more about pesticides in different areas and also to broaden society’s awareness of the^(17)^ problem.

In a study aimed at understanding the concept of health in public health, in the empirical material analysis, two polarizations were observed on the concept of health: on the one hand, the defense for constructing such a concept, existing within the study group several definitions about what health would be; on the other hand, the argument of the difficulty of conceptualizing health. It is noteworthy that collective health represents a social space in which critical analyses and relations between health and society^(18)^ are concentrated.

It can be said that the concept of health and its complex units, such as the human being or society, is multidimensional; in this way, the human being is at the same time biological, psychic, social, affective and rational. Relevant knowledge must recognize this multidimensional character and insert these data into it: not only could one not isolate a part of the whole, but the parts one from the other^(6)^.

When addressing education and health, it is considered that the way in which the topic “pesticide” is approached can influence how the citizen will think, act in society, for considering that information makes them capable of choosing what is good for themselves and recognizing themselves as an important and integral part of a questioning society. However, when individuals do not have the opportunity for critical reflection, especially because they are not aware of what is happening around them, directly or indirectly, they are influenced, considering only a social reality: that projected from other intentions^(19)^.

The great challenge of training for health education is precisely to educate for human understanding: teaching to be supportive, to be empathetic, to put oneself in the other’s shoes in a process of active listening. There are different perceptions built on the subject, and something that needs to be refuted is that health education aims only at the transmission of scientific knowledge, as this thinking, nowadays, is anachronistic. The importance of access to scientific knowledge that health education must provide is undeniable, but it must be considered that, by placing this purpose as the main one, other dimensions can be forgotten, such as co-responsibility, autonomy and the capacity of being cared for^(20)^.

Another important issue is the difficulty in identifying controversies and recognizing teachers’ and students’ common-sense view. The analysis of results in a given study sought to evidence whether the teachers were able to identify students’ explanatory limits in relation to contradictory situations evidenced in the data collected in the local reality study. It was highlighted that teachers cannot fully identify the vision they have of the social contradictions of their own world, which makes it difficult to understand students’ perceptions, and, therefore, dialogicity is not enough to solve the problems that have arisen, and it is necessary to strengthen the topics worked on and other educational aspects^(21)^.

In a study carried out with undergraduate education students in the countryside, it was shown that the topic of pesticides in schools is still incipient, and, due to its importance, as it is part of society reality, its systematic approach would be necessary. It was considered that, due to the fact that the topic of pesticides is controversial, teachers may have difficulties in discussing it in the classroom. However, it should be reported that it is necessary to approach controversial topics in the training space with different views so that, in this way, science teaching can favor students’ ability to argue and decision-making power, with analysis and critical positioning and social practice in the face of topics that interfere in their lives^(22)^.

It is considered that dealing with controversial topics in the classroom favors the greater insertion of students in social and techno-scientific issues, providing them with conditions for contextualized training of their points of view. To this end, it becomes important that, in the curriculum of science teaching, these topics are placed so that socio-scientific controversies that, in some way, impact individuals’ daily lives, are discussed and encouraged by teachers in discussions^(4)^.

Thus, the education of the future is confronted, because there is an increasingly wide inadequacy: on the one hand, disunited, divided, compartmentalized knowledge and, on the other, increasingly multidisciplinary, transversal, multidimensional, transnational, global and planetary realities and problems. In this inadequacy, one can cite as invisible: the context, the global, the multidimensional and the complex. Education must become evident in this way that knowledge is relevant^(23)^.

Thus, among the senses of seeing, experiencing and understanding the problems of our society, it is understood that, in the search for knowledge, self-observing activities must be inseparable from observing activities, self-criticism, inseparable from criticism, reflective processes, inseparable from objectification processes^(23)^.

In this regard, it is considered that the construction of knowledge requires clarity, requiring successive approximations and constructions, since we see a small part of the whole and what surrounds it, and, when we learn, we go through a period of reflection that is influenced by our contexts, experiences, previous knowledge. This movement shows us that we see a “drop in the ocean”, and, since this part presents limitations, our interest in knowing the reality of others must exist as a way of acquiring greater knowledge and proposing new shares aimed at joint growth^(6)^.

Finally, it is pointed out that it is necessary to strengthen the subjects involved in rural schools, such as students so that their opinions on the lived contexts are built together with their community so that controversies provide new possibilities. It is relevant to emphasize that controversies should not inhibit educators, but propose new possibilities, in which bridges are created between different knowledge, with challenges, new perspectives so that students feel included and encouraged to form their opinions, and, for this to exist, the environment must be conducive to dialogue and not judgment.

### Study limitations

A limitation is the sample of subjects in the research that focus on the regional context of western Paraná. However, it is important to emphasize that, in qualitative analyses, the aim is to value the complexity found in the subjects with their nuances and contradictions, making it possible to approach topics relevant to society from what is identified in small groups. Another challenging/limiting point in the study, given the importance of including the discussion on the topic in the school curriculum, is to make the topic attractive to adolescents, considering that this age group is constantly exposed to a large number of information and different topics.

### Contributions to nursing, health education, and public policies

The study sought to bring the concept of health closer to topics experienced daily in rural schools and, with this, to strengthen discussions in which health is something built by the community and strengthened every day in different contexts, demonstrating that falling ill is part of the vital process and does not represent health only as the absence of disease. It is considered that nursing, together with health education and the integration of educators and managers, can strengthen spaces in the community, such as schools, in order to strengthen public policies, rural population rights, community health in schools, among others.

## FINAL CONSIDERATIONS

The study strengthened the view that the topic of pesticides is of great relevance to the debate, and may be associated with health and the breadth of its concept, especially in rural schools, requiring greater emphasis by teachers so that it corresponds to a quality education for that space.

To this end, support from the school community is necessary, since teachers need to be strengthened within their spaces of action so that it is possible to overcome the institutional barriers, sometimes imposed, and, thus, to be closer to the rural community.

The aim is, therefore, to involve educators, students, families, health professionals and society in a broad way so that this construction is coherent in the face of the different paradigms faced, whether in the educational, social, health spheres, among others.

Students presented opinions that can be considered controversial, but the role of teachers is essential for them to understand this vision in a critical and contextualized way. Rural schools were identified as an important space for interaction of rural communities, considering that there are several social relationships that take place in their environment, in addition to contemplating family members, professionals, among others.

Finally, it is noteworthy that data construction was carried out before the pandemic, and the completion of the research, later, characterizing the experience of this study in a period of great fragility in world health. It is reinforced that, with the COVID-19 pandemic, environmental issues, such as destruction of natural environments, large-scale production, use of natural resources, among others, they need to be in focus both in the community and in the academic environment, since they are directly related to the population’s health.

Thus, health education should be valued as an important tool and connection between the university, rural schools and health services, being essential to generate new possibilities to face these problems, whether in the development of new research, integrated actions with the community and health services, for the support of universities with their projects and other possibilities.
